# Psychosocial strain and coping of Finnish working mothers during the COVID-19 lockdown: a job demand-control approach

**DOI:** 10.3389/fpubh.2024.1304319

**Published:** 2024-03-07

**Authors:** Venla Panula, Nelli Lyyra, Angeliki Kallitsoglou, Emmanuel Acquah, Pamela-Zoe Topalli

**Affiliations:** ^1^Department of Teacher Education, Faculty of Education, University of Turku, Turku, Finland; ^2^Health Promotion and Health Education, Faculty of Sport and Health Sciences, University of Jyväskylä, Jyväskylä, Finland; ^3^School of Education, University of Exeter, Exeter, United Kingdom; ^4^Faculty of Education and Welfare Studies, Åbo Akademi University, Turku, Finland

**Keywords:** COVID-19, mothers, job demand-control model, homeschool, work, stress

## Abstract

**Introduction:**

In March 2020 many countries around the world, including Finland, implemented lockdown measures to mitigate the unprecedented impacts of the coronavirus infectious disease (COVID-19) on public health. As a result, school and daycare settings closed indefinitely and working from home became the new normal for a big part of the workforce, which came with increased homeschooling and childcare responsibility for mothers.

**Methods:**

In this article we present the findings from maternal responses to open ended questions on psychosocial well-being, and experiences of combining work, family life and homeschooling during the COVID-19 national lockdown in Finland in March–May 2020. Working mothers’ responses (*n* = 72) were analyzed through the lens of Karasek’s job demand-control model, focusing on how the mothers experienced the demands of their life during the lockdown, and how they saw their possibilities to control the situation.

**Results:**

The findings indicated important variation in the level of experienced demand and control and associated compensatory factors during the COVID-19 lockdown across different subgroups of working mothers.

**Discussion:**

The findings have implications for understanding strain and plausible supports among working mothers during the COVID-19 lockdown as well as in the face of acute adversity including the next possible public health crisis.

## 1 Introduction

The beginning of the year 2020 brought a new set of demands into the lives of parents worldwide. When the COVID-19 pandemic hit, nations were forced to make decisions to restrict the contacts between people to control the spread of the virus. Among others, these restrictions affected the workforce, pupils, and students in all forms of education, as well as daycare possibilities for children.

In Finland, the government issued a state of emergency in March 2020. This lockdown caused by COVID-19 meant working from home for most adults, and attending school or studies remotely for children, adolescents, and young adults. During the lockdown only those who worked in societally essential roles were allowed to continue working at their workplace. In addition to schools, childcare services were also at first restricted to the children of these essential workers. This meant that both work, studies, and childcare all suddenly took place inside the home. The Finnish lockdown lasted from March 8, 2020, to May 13, 2020.

### 1.1 The effects of the restrictions to parents’ workload

As remote work and remote school became the new normal overnight, parents were faced with new challenges to keep the daily life of the family running while also taking care of their paid work. Before the pandemic mothers spent more time doing domestic work and caring for children than fathers [e.g., (1–3)]. During spring 2020, mothers spent even more time on childcare than before, despite doing more than their spouses already before the pandemic [e.g., (4, 5)].

Compared to pre-pandemic numbers, there was a significant increase in the hours men used for childcare during the pandemic (1, 4, 5). This meant that the relative gender gap in the hours devoted to childcare reduced during the pandemic [e.g., (1, 4)]. Despite that, in absolute numbers, women still put more hours into childcare and domestic tasks than men (1, 4). In addition, the women’s larger share of childcare was not explained by their level of employment. Only men who were furloughed did as many additional hours of childcare as women who were at work or worked from home (4).

Working mothers were more often the sole provider of care to their children, and more likely to become the sole provider of care during spring 2020 (5). In the US, Zamarro and Prados (5) found that working mothers were more likely to combine childcare and work during the pandemic than working fathers. According to the results of Craig and Churchill (1), in addition to childcare, women also spent statistically significantly more time on housework and household management during lockdown than men. However, it should be noted that compared to fathers, mothers were also more likely to reduce their work hours during the pandemic (5, 6). The reduction in mothers’ work hours was especially prevalent in families with children aged 1–5 and children aged 6–12 where both parents were employed (6).

In a Norwegian study, Thorsteinsen et al. (7) found that mothers’ well-being reduced statistically significantly during the lockdown period compared to before the lockdown and did not recover to the pre-pandemic levels after the lockdown (measured in June 2020). In the US, Miller et al. (8) studied mothers’ experiences of emotional connection during COVID-19 with both close-ended and open-ended survey questions. They found that for most mothers, the reduced social contacts were experienced as challenging, leading to negative emotions and adverse mental health impacts. However, Miller et al. (8) also reported some mothers experiencing the new normal as a positive change, and many mothers reported having made changes into their lives to better cope with the new demands during the pandemic.

In an Australian study, having other family members caring for one’s small children was associated with less parenting stress for both mothers and fathers (9). The lockdown period caused by COVID-19 took away the safety net from many parents and increased the stress related to childcare because it was not possible to get help from grandparents, other relatives or even friends.

However, another previous study conducted pre-pandemic in the US found that mothers experienced less parenting stress when the fathers took on more childcare tasks (10). Based on the already published results that report fathers spending more time on childcare during the lockdown compared to pre-pandemic times, it could be assumed that mothers’ parenting stress was lower during the lockdown if they could share the added duties with their partner compared to mothers who did not have this possibility during the lockdown period.

### 1.2 Psychosocial strain explained with Karasek’s job demand-control model

Karasek’s (11) job demand-control (JDC) model is widely used in research focusing on work-related stress and well-being. The model assumes that control buffers the impact of job demands on work-related stress and supports satisfaction with the opportunity to engage in challenging tasks and learn new skills (11).

The JDC model is two dimensional with both dimensions, job demand and job control, influencing the level of mental strain related to work and working conditions. Job demands refer to quantitative aspects such as workload, time pressure and phase of work (11). Emotional demands are also frequently analyzed aspects of job demand (12). The second dimension, job control, refers to the extent to which a person has decision latitude and decision authority over his/her work tasks and work in general (11).

Also, expansions have been suggested on the JDC model such as integrating resources, active coping and social support (13–15). The JDC model has been expanded into job demand-control-support model JDCS (14, 15) to account for the effect of social support at the workplace.

In previous research about the COVID-19 pandemic, Karasek’s model has been utilized mostly when investigating the strain experienced by healthcare workers and teachers [e.g., (16–18)]. The increased demands at work during the COVID-19 pandemic have been confirmed to predict increased feelings of burnout for Spanish teachers (17). Nurses who reported high levels of job demands but also high levels of control also experienced higher levels of personal accomplishment and resilience (18). Martí-González et al. (17) found that for Spanish teachers, job satisfaction during the COVID-19 pandemic was predicted by experienced social support. They discuss that the experienced social support helped the teachers cope with the changes and uncertainty the pandemic brought and made them more satisfied with their job even in the stressful, new situation. Similar link between job strain and burnout during the COVID-19 pandemic was found for nurses (18).

In addition, McLeish and Redshaw (19) reported that in their study, social support had a positive influence on maternal emotional wellbeing. In another study, McConnell et al. (20) found that parents who experienced strong social support reported lower levels of child difficulties. Social support in the form of open, non-judgmental discussion, and feeling heard, promoted the mothers’ feelings of connection and confidence (19). In addition, the demand-control model has been previously modified and used to examine domestic job strain [e.g., (21)] as well as work–family conflict and family-work facilitation (22).

### 1.3 Individuals’ reactions to changes

Becker et al. (23) considered the participants’ preferences to separate their work from their personal life (segmentation preferences) and found that the shift to remote work was experienced very differently based on the segmentation preferences of the participants (i.e., if they wanted to keep work and personal life completely apart or not). Understandably, the shift to remote work would not be ideal for someone preferring to separate their work from their personal life, since work would encroach on their personal space. Thus, the ways individuals cope with the changes to life during the lockdown are also a result of these personal preferences and tendencies.

Previous research has explored links between flexible work arrangements and work–family conflict already before the sudden need to switch to telecommuting because of the COVID-19 pandemic. Flexible work arrangements have been linked to less severe work interference with family (24). In addition, more than their actual use, the mere availability of flextime and flexplace was negatively associated with family interference with work (24). However, despite its positive sides, work flexibility can also be experienced as a source of stress and may deplete one’s resources, since it brings new demands for self-control and causes the work-family roles to blur (24). A previous study found that receiving social support from others and being highly proactive in one’s job were conducive to achieving the best possible work–life balance (25). Becker et al. (23) studied employees in different fields during COVID-19 and found that in their sample higher reported job control predicted less emotional exhaustion and better work-life balance.

In addition to the different ways of reacting to change, previous research has also shown that there are statistically significant differences between women and men in the ways they make decisions regarding their work and careers. When making decisions regarding their work, women considered family matters and the needs of other people in their life more often than men (26). Women worked less hours and changed jobs/careers more often than men to respond to their family’s needs (26). Women have also been found to give more emphasis on family responsibilities when they make decisions about work role entry, participation, and exit (27). In other words, family responsibilities affect the number of hours women devote to their job, but also their decision about quitting a job.

### 1.4 The current study

There has been a lot of research on strain and homeschooling during the COVID-19 period, but less research has been done on combining paid work, homeschooling, and domestic work from the mother’s perspective. Most of the research during the COVID-19 pandemic using Karasek’s job demand-control model has focused on healthcare workers and school personnel [e.g., (16–18)]. The current research brings new knowledge about the lockdown period by extending the JDC model to include not only paid work but also domestic work during the lockdown. The current research focuses on both the demands mothers experienced regarding their paid work during the lockdown and the demands they faced simultaneously in their personal life. Without time to prepare for it, the sudden change to remote work and homeschooling caused new challenges for mothers, and these experienced challenges and various ways to cope with them are the key focus points of this research. This study aims to investigate not only how mothers experienced the lockdown in terms of strain both about paid work and domestic work, but also how they reacted to this strain and adjusted their life to cope with the new demands better.

This study aims to answer the following research questions:

1. Which characteristics distinguish mothers in terms of strain and control combining work, children’s homeschooling, and domestic work?
2. What demands and possibilities for control did mothers experience in combining work and domestic work during the COVID-19 pandemic?
3. Were any background factors (e.g., number of children, children’s age, work situation) associated with belonging to these four, identified groups?

## 2 Materials and methods

### 2.1 Data collection

The data was gathered through a survey form on Webropol. The survey was available in Finnish and English, and it was mostly distributed to working and/or studying mothers in Finland. Initially, the decision was made to use convenience sampling, so three of the authors (VP, NL, & P-ZT) distributed the survey link to their own networks of mothers. However, many of the participating mothers approached the researchers asking permission to share the survey link to their own networks of working mothers as well as social media groups directed to mothers, so the data collection ended up snowballing. The data collection was surprisingly positively received, resulting in the survey reaching many times the planned number of respondents.

The primary focus was to reach working and studying mothers in Finland to capture the experiences of mothers during the first lockdown in Finland in March–May 2020. The Webropol form was opened on 13.5.2020, which was the last day of the daycare and school closure and was closed 08.06.2020.

### 2.2 Participants

During the data collection period, the survey form was filled by a total of 129 mothers. However, since the focus groups of the Pandemic Mood study were working and/or studying mothers, two participants were excluded from the original sample based on their work status (one on maternity leave, 1 a stay-at-home mom).

The current research focuses on working mothers in Finland. Of the 127 working and/or studying mothers, 92 resided in Finland at the time of data collection. The mothers residing outside of Finland (*N* = 34), as well as one mother who had not shared her country of residence were excluded from the original sample. In addition, the mothers who were studying but not working during spring 2020 (*N* = 20) were excluded from this sample. To conclude, the final sample for the current research is 72 working mothers residing in Finland.

### 2.3 Background variables

Among the background variables, the mothers were asked to share their age, work status (full-time, part-time, studying, or other) as well as the location where they worked during the state of emergency period (working / studying from home or outside the home). They were also asked to fill in the number of children and age of each of their children, as well as the workplace of their spouse (working / studying from home or outside the home), if applicable. Despite it being fruitful for the following analyses, the survey did not include questions about the socioeconomic status of the mothers or families.

### 2.4 Open-ended questions

The mothers’ experiences of the lockdown period were gathered through six open-ended questions. The first question covered the general mood and main thoughts and feelings during the lockdown. The second question explored the possible changes in the mothers’ workload, and the feelings caused by these changes. Next, the mothers were asked to describe possible changes in their social life, and their feelings regarding these changes during the state of emergency. Fourth, they were asked to describe the possible changes in their everyday life (e.g., routines, feeding the family, sleep quality and schedule, exercising, free time) and the feelings related to these changes. The fifth open-ended question covered the experiences of combining the different tasks and roles during the lockdown. We asked the mothers to describe how it felt to combine (remote) work, childcare, and children’s remote school (if applicable), what made it easier or harder to handle them all and what kind of thoughts and feelings the situation brought to them. Lastly, the mothers were asked to report how they felt about society starting to open again, and if the ending of lockdown affected their emotions or thoughts. In the end of the survey form they also had the possibility to freely add anything they wanted to share or clarify that was not asked or did not fit into the other open-ended questions. To give enough space for the mothers’ experiences, the character limit in these open-ended questions was set to 2,500 characters.

### 2.5 Qualitative analysis

The data analysis was conducted by the first two authors, V.P. and N.L. The analysis process started with reading through all the data. It was decided that the focus would be on the demands and possibilities for control the mothers described in their answers related to both their paid work and their childcare and domestic tasks. Both V.P. and N.L. then read through all the data again and grouped the participant mothers into four groups based on the demands and possibilities for control reflected in the answers.

All participant mothers described some demands they experienced brought on by the restrictions and new kind of life caused by COVID-19, with extensive variation in the degree of severity of the demands. Using Karasek’s model the mothers’ descriptions were grouped to high demands when mothers described experiencing the number of demands as very high and feeling burdened by the demands both at home and work. Often these descriptions also included mentions of the demands increasing significantly because of the COVID-19 restrictions. Grouped as low demands were the descriptions of less severe demands. In these descriptions there were positive considerations too, with the new normal bringing with it some positive changes in the mothers’ life too.

Like the reports of demands, also the mentions of mothers’ possibilities to control the situation varied noticeably. The descriptions grouped as low control were mostly very negative and included severe reports of straining to cope with the tasks both at home and at work. The difficulties of balancing the need to support children’s schooling with their own workload and finding time for household chores were interpreted as low control. Low control also included mentions of the ways of working changing significantly because of the pandemic. Descriptions in which the mothers reported finding solutions to organize their daily life in a new way during the restrictions caused by the COVID-19 pandemic were interpreted as having high control. Additionally, mothers’ accounts of being empathetic and forgiving towards themselves and lowering their standards towards childcare, work, and household upkeep were considered as high control. The grouping was first done by both researchers individually, and then they were discussed together.

After the first grouping round, the agreement rate between the two researchers regarding the grouping was 80.6%. The rest of the participants were assigned into a subgroup after a thorough discussion in which V.P. and N.L. talked through their observations and interpretations and came to an agreement about the suitable subgroups for each participant they initially disagreed on. Finally, to make sure there was a clear understanding of the subgroups, both V.P. and N.L. separately drafted descriptions of the four different groups. These descriptions were then discussed through and merged into the final subgroup descriptions.

### 2.6 Descriptives

Descriptive statistics (e.g., frequencies and percentages) were run to examine the demographic characteristics and responses to background variables in the total sample and in each mothers’ subgroup. No statistical testing was conducted due to the qualitative nature of the study.

### 2.7 Ethical issues

Participation was voluntary and all participants were legal adults. Active consent was collected from all participants and a data protection statement file was provided along with the questionnaire. This study followed the guidelines of The Finnish National Board on Research Integrity (28).

## 3 Results

The mean age of the participants was 38.86 years (sd 6.24 years). The youngest participant was 26 years old, and the oldest was 56 years old. 81% of the participants worked full time, and 78% worked at home during the state of emergency (lockdown). One fourth of the sample was the only adult in the household, the rest of the participants lived with a partner.

### 3.1 Characteristics of each subgroup based on demands and control

In Karasek’s job demand-control model both demands and control are considered as either high or low, resulting in four different categories based on the different combinations of the two dimensions. In this study, the mothers were categorized into four subgroups based on their descriptions of demands and decision latitude regarding not only their paid work, but also the other parental tasks and domestic work. Participants who described high demands and high levels of control were assigned to the *active* subgroup. Into the *high strain* subgroup belonged the mothers who reported a significant number of demands but not many ways to control those demands. When demands were low, but feelings of control were high, the mothers were assigned to the *low strain* subgroup. Finally, the participants whose descriptions included low levels of both demands and control belonged into the *passive* subgroup. Next, each of these subgroups are described in detail. Table 1 summarizes the main characteristics of each subgroup.

**Table 1:** Summary of the main characteristics of each subgroup.

	Demands low	Demands high
Control low	**Passive** Both positive and negative descriptions of the life during the lockdown, but quite mild demands Lacking a sense of control, the descriptions were characterized by a lack of ownership in the situation Waiting for things to change instead of actively pursuing changes to cope with the restrictions Social support talked about as things that were not possible without trying to find solutions or other ways to keep in contact	**High strain** Burdened by the grown number of demands at home and at work Feelings of hopelessness and frustration A sense of inadequacy related to both work and their role as a mother Lacking a sense of control Social life severely affected by the restrictions, reports of receiving very little social support
Control high	**Low strain** Lockdown brought some demands, but also several positive sides A sense of control over the daily life, making decisions to support the well-being of themselves and their family Forgiving and flexible outlook on the expectations towards work and home life Social life affected, but this change was not considered as very burdensome	**Active** Many demands, both related to work and the home life Sharing duties with a partner Adjusting routines, creating a new schedule Becoming more self-compassionate, forgiving towards themselves, lowering expectations Social support from family and friends using new ways of being in contact

#### 3.1.1 Active – finding new ways to keep the daily life organized

The mothers in the *active* subgroup expressed a lot of concerns and demands brought by the pandemic and the lockdown measures. They had a vast number of new demands brought by the day-care and school closures and changes in the ways of working. The new demands included working from home while supervising one’s children, which caused moments of needing to be in two places doing two different things at the same time. The mothers in the *active* subgroup also mentioned managing their own work was difficult during the lockdown, partly because the switch to remote work was so sudden, and partly because they had so many other responsibilities to take care of at home during their work hours.

*“Working from home is ideal only if one can concentrate on work. But, I had to help homeschooling and homepreschooling to get done, had to cook, do grocery orders, clean and do the dishes and laundry and on top of all this get my work done. I started to get very tired and started losing sleep.”* ID 100, 2 children, single mother, working from home, children at home.

However, mothers in the *active* subgroup also reported having ways to control the situation and to cope with these new demands. They had actively sought and found ways to organize their daily life to keep some sense of stability in the new, unstable situation.

*“Remote work and childcare have gone surprisingly well, but a lot of that is due to the fact that my husband is at home too and we have shared responsibilities. There have been occasional moments when the walls have felt like closing in when I've had to be in two places and responsibilities at once, but the fact that I can close the door and let someone else look after the kids during the working day helps a lot.”* ID 5, 2 children, both adults working from home, children at home.

In the *active* subgroup, common methods to cope with the demands were adjusting the family routines and making a new schedule to fit the new daily life better. The mothers felt the routines and schedules of the family had to be altered for the family to get through the days more smoothly.

*“We declared a schedule right away and got their opinions/approval. We implemented it the first day and adjusted it slightly where it didn’t work. And they got used to it really fast. That saved us a lot of hassle.”* ID 93, 2 children, both parents working from home, children at home.

Many of the mothers in the *active* subgroup also described how they purposefully chose to be more self-compassionate and to ease up on things like children’s screen time to cope with the new demands. They expressed a need to react to the restrictions by changing their own views. Their ways of coping with the new demands were being more forgiving towards themselves and expecting less from themselves as employees, mothers, and housekeepers. When everything happened inside the home, the mothers in the *active* subgroup realized the list of things they had to juggle within a day grew immensely. But compared to the *high strain* subgroup, mothers in the *active* subgroup reported finding ways to navigate the new situation and coped better with the new demands.

*“Working remotely with a daycare-age child has been challenging at times, but I have learned to be kind to myself. If one day I can't get work done as efficiently as I could in the office, it's not the end of the world, but my responsibilities include looking after the child alongside my work. Of course, the biggest help has been the fact that my child has a half-sister who goes to distance learning and with whom my child's time has been well spent. My child also spends 5 days every two weeks with her father, and the occasional change of scenery has helped both the child and myself. Maintaining routines (e.g. daily outdoor activities) and easing up on certain things, such as screen time and treats, have been key.”* ID 32, 2 children, working from home, spouse working outside the home, children at home.

Naturally, social life was very restricted during the lockdown period. Many mothers missed the opportunity to meet friends, get together with family members, let the children play with their grandparents, and to have informal coffee breaks with coworkers. However, mothers in the *active* subgroup had found new ways to keep in contact with their loved ones, for example by switching to video calls or meeting outside while keeping a safe distance. Many of them also reported that keeping touch with others over the phone (either through calls or instant messaging apps) felt good and helped to ease the stress, because it felt like they were not alone in the new, demanding situation.

*“It has been nice to see that through WhatsApp groups and even Facebook workout groups or international live lectures, there is a sense of "contact with others" – we share the same situation and its challenges all over the world.”* ID 77, 1 child, single mother, working from home, child at home.

#### 3.1.2 High strain – burdened under the demands and worries

The mothers in the *high strain* subgroup described a myriad of demands and worries regarding the pandemic and the strict restrictions during the lockdown. They were very burdened with the new challenges regarding childcare, children’s schooling, their own work and/or studies, and the routines of the household during the lockdown.

*“It was difficult to combine distance learning, my own and the children's, my own school was constantly suffering, tasks to be done at night when the children were asleep. More chores than before so it takes up a big chunk of working time. Part-time work ended when the company closed, so that's good as I wouldn't have time to do it anymore.”* ID 120, 2 children, single parent, working from home, children at home.

While they also described positive moments and experiences, most of their descriptions were negative, painting a picture of very strained and stressed mothers. The reports of the mothers in the *high strain* subgroup lacked a coherent sense of control regarding the new challenges. Instead, the mothers felt lost and burdened by all the tasks and demands they had.

*“Life has become a job. I wake up in the morning and get straight to work. From that moment on, I work almost around the clock. The days have included small breaks for food, snacks and walks. The evenings have gotten longer and sleep was worse than it is at the moment. Normal working hours are 8am to 4pm, followed by 2-3 hours of hobbies and family time.”* ID 80, 1 child, both adults working from home, child at home.

In the *high strain* subgroup, mothers often reported feeling hopeless and burdened under their never-ending to-do list. For them, the lockdown period and all the covid restrictions brought a considerable number of new demands into daily life. They felt frustrated because combining all their tasks and roles was incredibly difficult.

*“Too much of the time it's just awful, too demanding, nothing works properly when there doesn't seem to be enough time and resources for anything. Perhaps what makes it harder is that the children always rely on me and my much bigger role in terms of children, food and home care. It causes irritation, anger, frustration towards the rest of the family and myself. On the other hand, it's easy to stay at home, not having to move the kids anywhere on a certain schedule. With the children's daily routines, I've hit my head against the wall and become frustrated when I fail to create smooth routines. Getting outside, for example, has usually required a tiring fight and struggle, leaving everyone feeling bad. What has made the time easier is having our own yard to move around in, also free of fear. Tending the corners of the yard little by little has brought good mood.”* ID 72, 2 children, both adults working from home, children at home.

In the mothers’ answers, the struggles to cope with these new demands were also associated with a sense of inadequacy: not being a good enough mother or a good enough employee.

*“Very difficult. Feeling inadequate in professional as well as parent capacity. Feeling guilty and neglectful. My child has adjusted by playing on their own more but they are also confused that though we are at home we can't give the attention they are used to. Caring for child and home all day and working after the child is asleep feels like having two full time jobs.”* ID 3, 1 child, both parents working from home, child at home.

Some mothers also described feeling guilty about the choices they made regarding their children’s care during the lockdown period. They felt inferior as mothers because they had taken their children to the day-care during the lockdown period despite working from home. None of them reported having gotten any negative feedback from others regarding the decision to take the child to the day-care but they still felt uneasy about it. These mothers seemed to think they could not be good mothers if they could not work productively from home while caring for their children at the same time.

*“Combining work and taking my kid to day care has made me feel guilty, but otherwise has only doubled my commuting, which I feel is a good thing as I just consider it as exercise (we cycle/walk to the day care, I then come back home). The guilt comes from feeling a horrible mom for not feeling I could manage alone with my kid all week long or enjoy the time with them. I know I could not combine work and having the kid home, I would just "underachieve" both work and being there for my kid, creating nice enough tasks for him.”* ID 15, 1 child, single parent, working from home, child in daycare.

Compared to the *active* subgroup, mothers in the *high strain* group described their social life and social support being more severely affected by the covid restrictions. Of course, some mothers in the *high strain* subgroup reported not being negatively impacted by the restrictions of their social life. But in general, in the *high strain* subgroup, most of the descriptions of the experienced social life and social support were strongly negative.

*“I've only seen one friend, in the playground we've kept our distance from other kids and parents, we don't go into town with the child etc., I don't see my work colleagues when I work remotely – it's lonely! I only have time to myself when the child is asleep, as I don't want to ask for an outside babysitter at the moment. As a full-time single parent, I am responsible for the whole day-to-day running of the household anyway, which is of course sometimes a burden, but this corona situation has brought even more problems and pressure. I'm tired.”* ID 118, 1 child, single mother, working from home, child at daycare.

*“Daily encounters with good colleagues have disappeared, friends are struggling with the same problems and I don't want to burden them with my own worries. I experience loneliness from time to time and it lowers my mood.”* ID 39, 2 children, working from home, spouse working outside the home, children at home.

#### 3.1.3 Low strain – finding enjoyment in the new normal

In the *low strain* subgroup, the demands the mothers described were less severe and overwhelming than in the previous, *high strain* subgroup. Mothers in the *low strain* subgroup reported experiencing demands regarding the new way of life during the lockdown, but their descriptions were milder and more hopeful.

*“More having to check if homework etc. has been done by the children, more pressure on us parents to make sure our children are attending school by distance learning. Working outside the home and had more stress about children being at home. No hobbies for anyone in the family, no social contacts like before. Mood even improved because of less rush in everyday life.”* ID 73, 3 children, both parents working outside the home, 2 children at home & 1 at daycare.

They also found more positive sides in the new situation and seemed to enjoy the so-called new normal to some extent. Compared to life before the pandemic, the slower life brought by the lockdown was even seen as a welcomed change of pace.

*“Being a single parent is so hard, and I really needed the break that this situation gave me. It's like being on holiday when you don't have to be involved in anything. The social, "must be active" life has disappeared and it's acceptable to just be at home.”* ID 123, 1 child, working from home, child at home.

In the *low strain* subgroup, mothers also felt being in control of their life despite the changes brought by the pandemic and the restrictions during the lockdown. Despite the restrictions, they felt they were able to make decisions to support the well-being of themselves and their families.

*“Has been really good. Although I've had to spend time with child on schoolwork it's probably the same as normal homework, just at a different time of day. We've had more time as a family and more time to do nice things together. Also, enjoyed better sleep as not had to get up so early for school and work. Eating better as well as we can cook or choose what we like rather than surviving on snacks for me and school meals that are not always enjoyed by my child. I think my husband misses his office lunches though.”* ID 92, 1 child, both adults working from home, child at home.

Mothers in the *low strain* subgroup had a flexible and forgiving attitude towards the expectations they had for both themselves and their family as a whole. They described similar attitudes towards the changes in their work, too. In addition, they saw many positive sides in the changes of their mode of work: working from home meant additional time to do chores for example during coffee breaks, and not having to leave for the office helped them get more sleep and kept the mornings calmer.

*“It's been OK. I don't get stressed out if the kids miss a little something. They are self-directed and competent, and the teachers are great!”* ID 27, 3 children, both adults working from home, children at home.

Compared to mothers in the *high strain* subgroup, mothers in the *low strain* subgroup described less severe consequences to their social life. The lockdown affected the possibilities to meet friends, family, and coworkers face to face, but these changes did not seem unbearable. In addition, compared to the *high strain* subgroup, mothers in the *low strain* subgroup did not write about missing the possibility to have someone take care of their children for a moment.

*“Meeting friends partially interrupted. Lots of Teams meetings.”* ID 35, 4 children, both adults working outside the home, children at home.

*“Meeting less friends/family which of course hasn't been nice. But I have had to adapt to the situation and use my free time in other ways, e.g. a lot of outdoor activities and hiking.”* ID 119, 1 child, single mother, working outside the home, child outside the home.

#### 3.1.4 Passive – drifting forward

In the *passive* subgroup, the mothers reported moderate demands. They expressed both positive and negative emotions regarding their life during the lockdown, but the demands they experienced were quite mild.

*“Some changes in working practices, but not much. There has been a little less work in one job and a little more in another. Meeting friends completely on hold and I find myself missing it. Can't say I'm depressed yet, but if it goes on much longer, my mood will start to drop. I also miss my relatives. There's no time for my own hobbies after working and supervising distance learners. We go to the supermarket less often. A little more things have been done together with the family, but we did things together before as well.”* ID 69, 3 children, mother working outside the home, spouse working from home, children at home.

*“Routines have remained almost the same. The only things that have changed are longer days at work and the child's hobby has been left out.”* ID 24, 2 children, single parent, working outside the home, children in care outside the home.

Based on their answers, mothers in the *passive* subgroup seemed to be drifting forward in their daily life without a clear sense of control regarding their life events. Many of them seemed to have accepted the new demands and changes as given from above and being out of their control. The descriptions in the *passive* subgroup were characterized by a lack of ownership in the situation. Instead of pursuing control in the direction their life was going, the mothers just waited for things to change again.

*“I've experienced the fact that we've survived but hopefully it won't last long because life gets boring without other people.”* ID 116, 3 kids, both adults working from home, children at home.

Mothers in the *passive* subgroup described their situation during the lockdown as something that just happened to them. They viewed life during the restrictions as if they had no possibility to affect the situation or the circumstances they found themselves in. Compared to the *active* subgroup, mothers in the *passive* subgroup did not report many changes they would’ve done to cope with the new demands better.

*“A huge change. Less exercise, missing friends, just talking on the phone. It's exhausting and frustrating. There is a huge increase in housework and my husband has had less time to contribute. It frustrates me when people tell me how they get a lot of extra chores done at home and I can barely keep on top of them.”* ID 40, 3 children, both adults working from home, children at home.

This was true also for their answers regarding their social relationships and social support during the lockdown. In their answers, mothers in the *passive* subgroup described what was missing or what they had not done, while there were not many examples of things the mothers would have done to try to change things for themselves or their family.

*“I cannot meet my parents because they are in the risk group. Neither can they help with babysitting. I have not invited anyone home for 2 months, and have not visited anyone's house either. This is a big change to previous. Also when cafes and restaurants are closed it's hard to meet friends. I have met friends only outside (walks).”* ID 9, 1 child, single mother, working from home, child at home.

*“The meetings with friends are completely on hold and I find myself missing them. I can't say I'm depressed yet, but if it goes on for a long time, my mood will start to drop. I also miss my relatives.”* ID 69, 3 children, both adults working from home, children at home.

### 3.2 Background factors associated with the subgroups

The participants were assigned into different subgroups based on their answers regarding the experienced demands and possibilities to control the situation using Karasek’s JDC-model. Almost half of the mothers (46%) belonged in the *high strain* subgroup, and in the sample, there were mothers belonging to each of the four subgroups (*active* 21%*; low strain* 18%*, passive* 15%). Table 2 shows the descriptives of the entire sample as well as the same descriptives by subgroup. Mothers in the *high strain* subgroup were on average 34 years old, most had two children (children mean age 8 years), worked full time (82%) and were living in a two-adult family (73%). In the *active* subgroup, mothers were on average 39 years old, had two children (mean age 8 years), and worked from home (93%). Also, the majority (73%) of mothers in this group were living in a two-adult family. In the *low strain* subgroup participants were 40 years in average, had most commonly (54%) one child with mean age of 11 years. In this subgroup nearly 40% were working part time which was a higher proportion compared to the other subgroups (e.g., 3% in high strain subgroup). Also working outside the home was more common in the low strain subgroup compared to the other subgroups. In the *passive* subgroup, participants were on average 37 years old, with quite an even distribution in the number of children (27% one child, 36% two children, 36% three children; mean age 9 years). In regard to their work situation, around one third in the *passive* subgroup were working outside the home, which is a lower proportion compared to the *low strain* subgroup but higher compared to the *high strain* and *active* subgroups. In this subgroup, the majority (82%) were living with a partner.

**Table 2:** Descriptive statistics of working mothers in the four subgroups (*n* = 72).

	*High strain n* = 33 (46%)	*Active n* = 15 (21%)	*Low strain n* = 13 (18%)	*Passive n* = 11 (15%)	All *n* = 72 (100%)
Age	34 yrs	39 yrs	40 yrs	37 yrs	39 yrs
*Number of children*					
1 Child	30%	20%	54%	27%	32%
2 Children	55%	60%	15%	36%	46%
3 Children	12%	20%	23%	36%	19%
4 Children	3%	0%	8%	0%	3%
*Age (children)*					
Mean age	7.69	8.27	10.71	8.65	9.00
Below 7 yrs	42%	37%	29%	43%	39%
7 to 15 yrs	55%	63%	50%	43%	54%
16 yr. +	3%	0%	21%	13%	7%
*Work*					
Full time	82%	100%	62%	73%	81%
Part-time	18%	–	39%	27%	19%
*Workplace*					
Home	85%	93%	54%	64%	78%
Outside the home	15%	7%	46%	36%	22%
*Family structure*					
The only adult	27%	27%	25%	18%	25%
Living with a partner	73%	73%	75%	82%	75%

In summary, mothers in two subgroups associated with high demands (*high strain, active*) were mostly working from home and had a full-time job. In comparison, subgroups associated with low demands (*low strain, passive*) were characterized with mothers also working part-time and outside the home. Instead, the proportions of one adult and two adult families were nearly the same in all four subgroups.

## 4 Discussion

This study focused on the experiences of mothers during the lockdown caused by the COVID-19 pandemic in Finland. The study examined the challenges and difficulties that working mothers experienced during the lockdown and the means they used to cope better with their new daily lives. The job demand-control model (11), originally developed for the work environment, was used as a framework for this analysis. In this study, the job demand-control model was modified to include not only work-related demands, but also demands related to childcare, children’s remote schooling, running the household during the pandemic and anything else directly linked to the daily life of the respondents.

### 4.1 Same external factors – different ways of reacting and coping

The premise of the study was that the restrictions associated with the COVID-19 pandemic and the resulting external, social changes were the same for all mothers. We were interested in seeing how Finnish mothers would report having experienced these restrictions and challenges. Instead of receiving reports of identical experiences, differences were seen between mothers in the way they reacted and adapted to these changes. Adapting to the restrictions and coping with new demands seemed to depend more on each mother’s individual resources, tendencies and, for example, the existence of a functioning, supportive social network.

These differences also link to the family resilience theory [e.g., (29)]. According to the family resilience theory, families differ for example in their outlook towards adversity, connectedness and support, open emotional expression, and collaborative problem-solving (29). In other words, the strain mothers experienced during the lockdown could be partly explained by family-level factors of resilience or coping, and not just the individual qualities of the mothers themselves.

### 4.2 Karasek’s Job demand-control model as a framework

Karasek’s (11) job demand-control model has previously been applied to the context of home, focusing on strain experienced at home. Based on earlier research, the JDC model (or JDCS, with social support added) is suitable to assess the experienced strain also at home, with regards to the demands and control related to not only work but also all the other responsibilities of one’s daily life [e.g., (21, 22)].

In this study, Karasek’s (11) model was used to classify mothers into four different groups according to the demands of their everyday life during the lockdown caused by COVID-19, and the mothers’ perceived ability to control their situation. Karasek’s model was well suited to the analysis of the data. In their responses, mothers reflected a considerable amount both on the pressures and accumulated demands they experienced and on the ways they coped with these new challenges. Based on the results, it can be said that the JDCS model is suitable for studying not only the strain experienced in the paid work context, but also in the context of one’s home and family life. In addition, considering the unique situation of the restrictions on social life brought by the COVID-19 pandemic, the social support aspect of the JDCS model is a well justified and necessary addition to the model in this case.

### 4.3 Main findings

The study set out to investigate the demands and possibilities for control mothers experienced in combining work and domestic work during the COVID-19 pandemic. In general, mothers’ responses highlighted the fact that the lockdown caused by the covid pandemic significantly increased their workload. They had to play the role of nanny, housekeeper, employee, and teacher, and many reported that they felt that there were not enough hours in the day to fulfil all these roles. This reflects the findings of Zamarro and Prados (5), who reported that in the US, working mothers were more likely to combine childcare and work during the pandemic than working fathers. Despite the relative gender gap in the number of hours put into childcare and household tasks reduced during the pandemic, women still put in more hours in those tasks than men (1, 4). The gender gap might explain why so many mothers felt so burdened during the COVID-19 lockdown period in Finland too.

Another possible factor explaining the burden experienced by mothers could be the fact that women emphasize family responsibilities more than men when considering work-related decisions like the amount of hours worked (26, 27). Therefore, the changes in the family life caused by the pandemic might have affected mothers more strongly because of this tendency to consider the needs of the family when making work-related decisions.

However, many mothers also felt that they could manage this new situation. They described how they had rearranged their daily routines and home spaces so that everyone had the best possible chance to concentrate on work or studying at home. Many had found it helpful to be able to share childcare responsibilities with their spouse, taking turns focusing more on work and then going back to childcare. On the other hand, those who continued to work outside the home had particularly little time to support and help their children during their distance learning days. There was considerable variation in the control and coping methods reported by the mothers. Some mothers reported being in control, taking charge and making changes in their life to cope better in the new situation. Others felt that they had neither the opportunity nor the resources to influence the situation, and that the demands of the lockdown felt overwhelming.

Among the participants, the variability in how they experienced the demands and their possibility to influence the situation meant that we found mothers belonging in all four groups based on the JDCS model. Almost half of the mothers (46%) were classified into the *high strain* subgroup, in which the increased demands of the lockdown were felt to be very burdensome, and they felt they had little control over the situation. Mothers in the *active* subgroup (21%) did experience a lot of demands and difficulties, but they had also found new ways to cope with them. Their experience was accentuated by a sense of control, having managed to structure their new daily lives so that they felt in control.

Both in the *low strain* and *passive* subgroups, mothers felt that the demands of both work and everyday household life were generally quite moderate. The mothers in the *low strain* subgroup (18%) also found positive aspects of this new daily life and seemed to perceive the new way of life during the lockdown positively. The responses of mothers in the *passive* subgroup (15%) highlighted the lack of a sense of control. They experienced some demands, but clearly less than mothers in the *high strain* subgroup. Compared to the *low strain* subgroup, mothers in the *passive* subgroup felt that they had little control over their situation. Rather, they took the challenges as they came and did not feel in control of them or their new daily lives.

### 4.4 Demands and strain related to employment and keeping the home life going

According to a report by the Finnish government, while working from home in 2021, women were more likely to work in a space that was also used for something else too (e.g., by the kitchen table) than men (30). These differences between men and women were even bigger in families in which both parents worked from home. This was seen in our results too. Mothers described how difficult it was to focus on work while also juggling childcare, children’s remote schooling, cooking and feeding the family, taking care of household chores, and trying to maintain social relationships outside the family too. This seems to reflect the tendency of women to consider the needs of their near and dear, and to make decisions about reducing their work hours to have more time for their family (26, 27). The participants tried to do what was best for their family while also trying to keep working as productively as possible, which clearly was a very difficult combination during the lockdown. In addition, while flexible work arrangements like telecommuting are associated with less work interference with family, flexibility can also be a source of stress and strain, for example because it might cause blurring of the work and family roles (24).

The level of difficulty and strain was highlighted in some mothers’ descriptions in which they mentioned feeling guilty for making decisions during the lockdown based on their own needs. Some mothers reported feeling guilty because they could not work productively at home while the children were at home too. They ended up taking the child (ren) to the daycare during the lockdown to be able to continue working from home and felt like bad mothers for doing so.

For some mothers, the restrictions brought a welcome opportunity to spend more time with the family and take a break from other obligations. This was especially true for mothers in the *low strain* group. They reported having enjoyed the possibility to stay at home and not run around from one place to another day in day out. This echoes the findings reported by Miller et al. (8), who found that for some mothers the restrictions offered a welcomed chance to live a quieter life with less obligations to partake in activities outside the home.

### 4.5 Social life

Regarding the ways the mothers coped with the new demands, especially mothers in the *active* subgroup described having worked on finding new ways to maintain social relationships with loved ones. They reported how sharing their experiences and feelings with friends and family helped to ease the stress in the new, very demanding situation. This is in line with the results of McLeish and Redshaw (19), who reported that social support (open, non-judgmental discussion and feeling heard) promoted mothers’ confidence and feelings of connection to others. This reflects the previous results of Miller et al. (8), which highlighted that open, empathetic, and supportive conversations with friends and family strengthened the emotional connections mothers felt with their loved ones during the COVID-19 pandemic.

Mothers in the *high strain* subgroup reported severe effects on their social life: they expressed having lost connection to others, not having time to maintain social relations, and even chose to withdraw from social interactions because they did not want to burden their friends and family further with their own worries. These findings link to the results of Miller et al. (8), who found that mothers who reported the biggest decreases in social contacts felt being more physically and socially distanced from others.

Social support has also been found to be associated with a better work–life balance [e.g., (25)]. Having supportive relationships with one’s colleagues even during the lockdown and everyone working from home helps to keep the balance between one’s work and family life.

### 4.6 Individual resources and social networks meaningful explanatory factors for experienced strain

Based on our data, it cannot be said that any single background factor intensified the experience of strain or led to belonging to a particular subgroup. For example, there was variation in the average age of children between subgroups, but the differences were not statistically significant. In addition, regarding relationship status, one might have predicted that single mothers, for example, would have experienced the lockdown as the most burdensome and that the *high strain* subgroup would have had a higher proportion of single mothers than the other groups. However, this was not the case.

Adapting to the restrictions and coping with new demands depended on each mother’s individual resources, tendencies, and social networks. In other words, the number of children, their age or, for example, the relationship status of the mother did not explain the way in which mothers experienced strain during the lockdown. More relevant to the experienced strain were the quality of the possible romantic relationship, the degree of involvement of the spouse in the family’s daily life, and the quality of social networks and the support received through them. Considering having family members care for one’s child being associated with less parenting stress (9), the support received from one’s social networks during a pandemic plays a big role in mothers’ well-being. Additionally, the individual resources and coping skills of the mothers were noticeably different between the different subgroups.

The sudden shift to remote work was easier for some than others. The differences in how it was received were clear in the mothers’ descriptions: some were happy to have more time at home and not having to rush to the office, others were struggling with finding the space and time to allocate for paid work while trying to stay on top of household chores too. These struggles could partly be explained by the personal segmentation preferences, meaning how important it is for one to keep work and personal life separated (23). For mothers who consider it important to keep work completely separated from their family life, having to work from home could mean a serious struggle, because that segmentation is no longer possible as they would prefer. While Allen et al. (24) reported that flexible work arrangements like telecommuting and flextime were associated with less severe work interference with family, the negative sides of this flexibility should also be considered. Not everyone enjoys bringing their work home, and based on our results this blurring of the boundaries of work and personal life can make life very difficult for some individuals.

### 4.7 Strengths and limitations

The lockdown during the spring of 2020 due to the COVID-19 pandemic provided an exceptional setting to study that would not be possible under normal circumstances. Such extensive restrictions on people’s daily lives and social interactions cannot simply be imposed on a whim, and opportunities to study such situations do not arise at any time.

One of the strengths of this study is the breadth of the data. We offered mothers the opportunity to share their experiences in their own words, and most of the participants took advantage of this opportunity. A large proportion of the mothers’ responses were long, they had clearly taken a lot of time and had considered the issue from many angles. Respondents seemed to have found it meaningful to participate in the survey. Reaching our target group also proved easy, as mothers saw responding to the survey as a good opportunity to reflect on their own situation, and many also felt it important to pass on the link to their close contacts.

Conversely, the study is limited by the relatively small sample size, which means that the results cannot be generalized to a larger sample. However, the qualitative approach used serves to justify the small sample size. The small size of the data also limited the ways in which subgroups could be compared with each other on different background variables. Additionally, as mentioned in the methods section, the survey did not include questions about the participants’ socioeconomic status or level of education, for example, which limits the conclusions that can be made based on the study. A similar study design but with a larger data set would work well as follow-up research. Then, in addition to the grouping based on Karasek’s model, the subgroups formed could also be compared more closely with each other, to find factors that would explain membership of each group statistically significantly.

## 5 Conclusion

Instead of background factors, adapting to the restrictions and coping with the new demands brought on by the COVID-19 pandemic depended on each mother’s individual resources, tendencies, and social networks. The experienced strain is not only due to external factors like not having a partner or having multiple young children, but also due to individual factors. Therefore, it is important to not make assumptions about an individual’s experienced stress or strain based on external factors. Instead, the focus should be on the actual emotions and experiences of the individuals, and to achieve that, there should be an opportunity for everyone to be heard.

## Data availability statement

The datasets presented in this article are not readily available because of the qualitative content of the data. Requests to access the datasets should be directed to VP, vspanu@utu.fi.

## Ethics statement

The requirement of ethical approval was waived by the Ethics Committee for Human Sciences at the University of Turku, Humanities and Social Sciences Division for the studies involving humans because the Ethics Committee for Human Sciences at the University of Turku, Humanities and Social Sciences Division. The studies were conducted in accordance with the local legislation and institutional requirements. The participants provided their written informed consent to participate in this study.

## Author contributions

VP: Conceptualization, Data curation, Formal analysis, Investigation, Methodology, Visualization, Writing – original draft, Writing – review & editing. NL: Conceptualization, Formal analysis, Investigation, Methodology, Writing – original draft, Writing – review & editing. AK: Writing – review & editing. EA: Writing – review & editing. P-ZT: Conceptualization, Writing – review & editing.
